# Comparison of High-Dose Cytarabine, Mitoxantrone, and Pegaspargase (HAM-pegA) to High-Dose Cytarabine, Mitoxantrone, Cladribine, and Filgrastim (CLAG-M) as First-Line Salvage Cytotoxic Chemotherapy for Relapsed/Refractory Acute Myeloid Leukemia

**DOI:** 10.3390/jcm9020536

**Published:** 2020-02-16

**Authors:** Ciera L. Patzke, Alison P. Duffy, Vu H. Duong, Firas El Chaer, James A. Trovato, Maria R. Baer, Søren M. Bentzen, Ashkan Emadi

**Affiliations:** 1 Greenebaum Comprehensive Cancer Center, University of Maryland, Baltimore, MD 21201, USA; Ciera.Patzke@umm.edu (C.L.P.); aduffy@rx.umaryland.edu (A.P.D.); mbaer@umm.edu (M.R.B.); sbentzen@som.umaryland.edu (S.M.B.); 2 School of Pharmacy, University of Maryland, Baltimore, MD 21201, USA; jtrovato@rx.umaryland.edu; 3 School of Medicine, University of Maryland, Baltimore, MD 21201, USA; 4 School of Medicine, University of Virginia, Charlottesville, VA 22908, USA

**Keywords:** acute myeloid leukemia, relapsed, refractory, CLAG-M, HAM-pegA, asparaginase

## Abstract

Currently, no standard of care exists for the treatment of relapsed or refractory acute myeloid leukemia (AML). We present our institutional experience with using either CLAG-M or HAM-pegA, a novel regimen that includes pegaspargase. This is a retrospective comparison of 34 patients receiving CLAG-M and 10 receiving HAM-pegA as first salvage cytotoxic chemotherapy in the relapsed or refractory setting. Composite complete response rates were 47.1% for CLAG-M and 90% for HAM-pegA (*p* = 0.027). Event-free survival was significantly different in favor of HAM-pegA (*p* = 0.045), though overall survival was similar between groups. There were no significant differences in toxicities experienced by patients treated with the two regimens, including adverse events of special interest related to pegaspargase (venous thromboembolism, hemorrhage, hepatotoxicity, pancreatitis, and hypersensitivity reactions). HAM-pegA is a novel regimen for relapsed or refractory AML that resulted in improved response rates and similar toxicities compared to CLAG-M.

## 1. Introduction

Standard induction therapy for patients with newly diagnosed acute myeloid leukemia (AML) with a good performance status typically includes cytarabine plus an anthracycline [1]. However, about one-third of patients will not respond, and 50% or more of patients who do respond will eventually relapse [2]. Prognosis remains poor for patients with relapsed or refractory (R/R) AML, with median survival from relapse of approximately 6 months, and 2- and 5-year survival estimated at 16% and 10%, respectively [3]. Currently, no standard of care exists for the treatment of R/R AML, particularly for patients with good performance status who lack mutations that may predict for response to targeted therapy [1].

Two intensive salvage regimens, CLAG-M (cladribine, high-dose cytarabine, filgrastim, mitoxantrone) and HAM-pegA (high-dose cytarabine, mitoxantrone, pegaspargase), are commonly used at our institution (outside of clinical trials) for the treatment of R/R AML, based on physician preference. CLAG-M was studied in a prospective Phase II trial, demonstrating a complete response rate of 58% and a median overall survival of 9 months [4] and is currently listed as an option for R/R AML in the National Comprehensive Cancer Network (NCCN) guidelines [1]. HAM-A (high-dose cytarabine, mitoxantrone, short-acting L-asparaginase) is supported by a single-arm retrospective study that showed a response rate of 41% and a median overall survival of 6.1 months [5]. HAM-A is unique in that it incorporates short-acting asparaginase, a drug normally used for acute lymphoblastic leukemia (ALL) rather than AML. It is postulated that asparaginase may also have activity against myeloid leukemia cells through mechanisms including plasma depletion of both asparagine and glutamine [6]. To date, there are no published data comparing these two regimens. Additionally, HAM-pegA, substituting pegylated asparaginase (pegA) for L-asparaginase in HAM-A, has not been reported in the literature, but is in common use at our institution due to the lack of commercial availability of short-acting L-asparaginase in the United States.

The purpose of this study is to compare the efficacy, safety, and tolerability of HAM-pegA versus CLAG-M as first-line salvage cytotoxic therapy for R/R AML. We particularly aimed to describe the adverse event profile of HAM-pegA, given the lack of current literature describing its toxicities.

## 2. Experimental Section

This was a retrospective cohort study of consecutive patients who met the inclusion criteria below, receiving either HAM-pegA or CLAG-M as first-line salvage cytotoxic chemotherapy for R/R AML between 1 March, 2012 and 8 April, 2019 at the University of Maryland Greenebaum Comprehensive Cancer Center (UMGCCC). The primary endpoint of this study was to compare the composite response rates (complete remission with or without complete count recovery) between patients receiving HAM-pegA versus CLAG-M. Secondary endpoints were to compare: (1) complete remission (CR) rates, (2) 30-day, 60-day, and overall survival, (3) event-free survival (EFS), (4) length of hospital stay, (5) incidence of subsequent hospitalization, and (6) incidence of ≥grade 3 adverse events as per Common Terminology Criteria for Adverse Events (CTCAE), including adverse events of special interest related to pegaspargase. This study was approved by the University of Maryland, Baltimore Institutional Review Board (IRB).

Patients were retrospectively identified for inclusion in our study based on chemotherapy regimens received during the specified study time frame. Patients were included if they were 18 years or older and had relapsed or refractory AML documented by bone marrow aspirate and biopsy. AML was defined by World Health Organization (WHO) 2018 criteria, and the diagnosis was confirmed by a pathologist at our institution.

CLAG-M and HAM-pegA were used concurrently at our institution based on physician preference. Patients treated with CLAG-M received TBO-filgrastim 300 mcg subcutaneously starting the day prior to chemotherapy (day 0) and continuing through the last day of chemotherapy (day 5), cladribine 5 mg/m^2^ intravenously (IV) over 2 h daily on days 1 through 5, cytarabine 2000 mg/m^2^ IV over 4 h daily on days 1 through 5 (given after cladribine, with a cumulative cycle dose of 10 g/m^2^), and mitoxantrone 10 mg/m^2^ IV over 30 min daily on days 1 through 3. Patients treated with HAM-pegA received cytarabine 3000 mg/m^2^ IV over 3 h every 12 h for 5 doses (days 1 through 3, with a cumulative cycle dose of 15 g/m^2^), mitoxantrone 6 mg/m^2^ IV over 60 min immediately following doses 1, 3, and 5 of cytarabine (days 1 through 3), and pegaspargase 2500 units/m^2^ IV (with no dose capping) over 2 h on day 4. Cytarabine could be dose-reduced to 1000 mg/m^2^ in both regimens based on age (>60 years) and renal function (serum creatinine 1.5–1.9 mg/dL) of the patient, resulting in a cumulative cycle dose of 5 g/m^2^ for both regimens. All intravenous chemotherapy was administered via central access. Patients were not treated with either regimen if their serum creatinine was ≥2 mg/dL. Patients were assessed for study outcomes after a single course of induction therapy.

Baseline data collected included age, sex, race, French-American-British (FAB) AML subtype, primary refractory disease or relapsed disease, Eastern Cooperative Oncology Group (ECOG) performance status, previous chemotherapy or allogeneic hematopoietic stem cell transplant (allo-HSCT), percent bone marrow blasts, karyotype, and myeloid mutations. Outcome data collected included platelet and neutrophil nadirs, time to platelet and neutrophil recovery from day 1 of chemotherapy, bone marrow aspirate and biopsy results after count recovery, length of hospital stay, duration of CR/CRi (if achieved), subsequent hospitalizations, event-free survival (defined as time to either relapse or death), overall survival, and adverse events associated with each regimen. CR was defined as <5% bone marrow blasts, no evidence of extramedullary disease, absolute neutrophil count (ANC) ≥1000/mcL, platelets ≥100,000/mcL, and red blood transfusion independence. Complete remission with partial count recovery (CRh) is defined as <5% bone marrow blasts and no evidence of extramedullary disease with platelet count >50,000/mcL and ANC >500/mcL. Complete remission with incomplete count recovery (CRi) is defined as <5% bone marrow blasts and no evidence of extramedullary disease with either platelet count >100,000/mcL or ANC >1000/mcL but not both. 

For 2-by-2 tables, Fisher’s exact test was used to test for independence. For other contingency tables, Pearson’s Chi-Square test was used. For multi-level ordinal and continuous data, the non-parametric Mann-Whitney U test was used. Overall survival (OS) was calculated as the time from cycle 1 day 1 of CLAG-M or HAM-pegA until the date of death from any cause. Patients who were still alive at the date when the data set was locked for analysis were censored on that date. EFS was calculated from the date of first treatment until the date of relapse, progression or death. Data were censored at the date of last follow-up. Time-to-event data were estimated using the product limit (Kaplan-Meier) method, with the Mantel-Cox log rank test used to compare hazard rates in the two treatment groups. All *p*-values are two-tailed except when otherwise specified. A *p*-value of 0.05 or less was considered statistically significant.

## 3. Results

A total of 54 patients were identified. Five patients were excluded for having mixed phenotype acute leukemia and three patients for having acute lymphoblastic leukemia. Two patients receiving CLAG-M were also excluded for not receiving all of the planned chemotherapy doses (one died after the first day of chemotherapy, and one switched to decitabine before completing CLAG-M). The final study population included 34 patients treated with CLAG-M and 10 patients treated with HAM-pegA. Baseline demographics are listed in Table 1. All characteristics were similar between the two treatment groups except race, with higher percentages of White and Black patients in the CLAG-M group and a higher percentage of Asian patients in the HAM-pegA group. Median age for CLAG-M and HAM-pegA groups was 52 and 49 years, respectively. A large majority of patients had good to excellent performance status (84.1%) and had received 7 + 3 as initial induction therapy (86.4%). One patient in the CLAG-M group previously received CLAG-M for initial induction, achieved an excellent complete response duration of 52 months, and received CLAG-M again upon first relapse. CLAG-M or HAM-pegA was the first cytotoxic regimen received for relapsed or refractory disease for all patients, but a few patients did receive a non-cytotoxic therapy such as DNA methyltransferase inhibitors for first relapse (10.0% for the HAM-pegA group and 11.8% for the CLAG-M group) prior to intensive re-induction. More patients had relapsed disease (63.6%) than refractory disease (36.4%). Three patients in the CLAG-M group and one patient in the HAM-pegA group received a reduced dose of cytarabine (1000 mg/m^2^ per dose) based on age and/or renal function.

**Figure 1:**
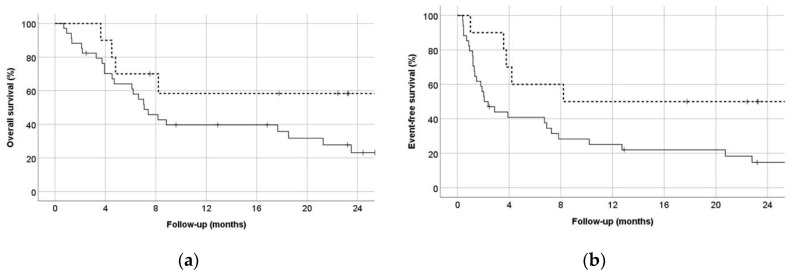
(**a**) Overall survival, with the solid line representing CLAG-M and dashed line representing HAM-pegA (*p* = 0.135) (**b**) Event-free survival, defined as time to relapse or death, with the solid line representing CLAG-M and dashed line representing HAM-pegA (*p* = 0.045).

**Figure 2:**
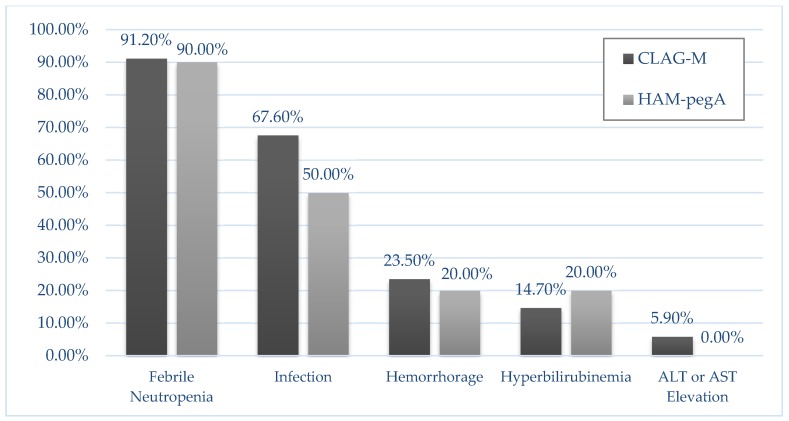
Incidence of grade 3/4 Adverse Events in 30 days. No adverse event was statistically different between the two groups. There was an absence of the following grade 3/4 adverse events in both groups: venous thromboembolism, alkaline phosphatase elevation, hepatic failure, amylase/lipase elevations, pancreatitis, and hypersensitivity reactions.

For the primary endpoint, composite complete response rates were 90.0% for HAM-pegA and 47.1% for CLAG-M (*p* = 0.027) (Table 2). Fifty percent of the patients in HAM-pegA and 26.5% in CLAG-M proceeded to allogeneic stem cell transplant. Median length of stay was 30 days and 36 days for HAM-pegA and CLAG-M, respectively. Mean numbers of re-hospitalizations within 6 months were between 1 and 2 for both groups. All patients treated with HAM-pegA survived to 30 and 60 days. In comparison, CLAG-M survival rates were 94.1% and 88.2% at 30 and 60 days, respectively. Overall survival at 12 months was not statistically significant between groups (*p* = 0.135), but EFS significantly favored HAM-pegA (*p* = 0.045) (Figure 1). Documented grade 3 or 4 adverse events were similar between groups (Figure 2). Febrile neutropenia and confirmed infection were the most common adverse events. Myelosuppression was profound in both groups, with median ANC nadir of zero in both groups, and median time to ANC recovery of 32 days and 38 days for HAM-pegA and CLAG-M responders, respectively. Similarly, the median platelet nadir was 6000/mcL in the HAM-pegA group and 5000/mcL in the CLAG-M group, and median time to recovery of 31 and 35 days, respectively. Transfusion of red blood cells and platelets were goal-oriented (e.g., platelets < 10,000/mcL, hemoglobin < 7–8 g/dL), and transfusion dependence was similar between groups. No grade 3 or 4 venous thromboembolism, alkaline phosphatase elevation, hepatic failure, amylase or lipase elevations, pancreatitis, or hypersensitivity reactions occurred in either group within the first 30 days. In the HAM-pegA arm, two patients had a grade 1 or 2 venous thromboembolism, one of which was catheter-related.

## 4. Discussion

The present study is the first to compare the HAM-pegA and CLAG-M regimens as initial cytotoxic salvage therapy for R/R AML. Additionally, it adds our institution’s experience with HAM-pegA to the limited literature currently available regarding this regimen. HAM-pegA is a unique regimen for AML that leverages the synergy postulated to occur between high-dose cytarabine and asparaginase when given sequentially [7]. Asparaginase appears to enhance the anabolism of cytarabine by reducing deoxycytidine triphosphate pools and thereby maximizing the therapeutic index of cytarabine. When added to high-dose cytarabine, asparaginase significantly increased complete remission rates and overall survival in adult patients with R/R AML when compared to high-dose cytarabine alone [7]. Additionally, asparaginase has been shown to have particularly high activity in AML when cell lines have a deletion of chromosome 7 [8]. Chromosome 7 is responsible for the production of asparagine synthetase, and its depletion (through a deletion of chromosome 7) results in the leukemic cell’s inability to overcome the activity of asparaginase. In the present study, there was only one patient in each group that had a deletion of chromosome 7, though the patient receiving HAM-pegA did achieve a complete response. 

The response rate for HAM-pegA in our study was notably high. The previous report of HAM-A had an overall composite response rate of 41%. However, this was highly influenced by the lower response rate seen in patients age 60 years and older (33%) which made up 70% of the study population [5]. Patients under the age of 60 years had a response rate of 61%, which is similar to the age of patients treated in our study. Response rates for CLAG-M in the present study were similar to what has been previously reported in the relapsed/refractory setting [4].

The median age of patients receiving HAM-pegA was above 40 years old in the present study. This differs from the typical use of asparaginase-based products in other hematologic malignancies, which is typically limited to children, adolescents, and young adults (age <40 years old), given the decreased tolerability of the toxicities associated with asparaginase and related products in older patients. Our toxicity data support the safety of using pegaspargase as part of the HAM-pegA regimen in an adult population (age >40 years old), as there were no clinically significant differences in adverse events when compared to CLAG-M. Additionally, the literature currently available regarding pegaspargase-related toxicities is limited to the ALL population, however it is possible that the toxicity profile may be different in an AML population. Our institution has recently developed and implemented guidelines for the prevention, monitoring, and management of pegaspargase-related adverse events to further ensure tolerability of pegaspargase in adults, including older adults [9]. Of note, since the adoption of these institutional pegaspargase guidelines, our institution now limits the pegaspargase dose to a maximum of 3750 units for all patients >18 years old. It is noted that dose capping may be more prudent in regimens for acute lymphoblastic leukemia, where multiple doses of pegaspargase are received. Patients included in the present study all received therapy prior to the initiation of these guidelines and received the 2500 units/m^2^ dose without dose capping.

Due to a lack of commercial availability of L-asparaginase, our institution substituted pegaspargase 2500 units/m^2^ on day 4 for the originally studied L-asparaginase 6000 units/m^2^ as a single dose on day 4 [5]. It is known that the pharmacokinetic profiles of pegaspargase and L-asparaginase are vastly different, with one dose of pegaspargase having a similar duration of effect as six to nine doses of L-asparaginase [10]. However, despite the increased exposure to asparaginase in the present study, clinically significant adverse events related to pegaspargase (i.e., venous thromboembolism, hemorrhage, hepatic failure, pancreatitis, and hypersensitivity reactions) were not statistically different from those seen with CLAG-M, and the response rate to this potent regimen was promising. Notably, patients receiving HAM-pegA in our study had a median age of 48 years (maximum age 61 years) compared to a median of >60 years in the study by Ahmed, et al. [5]. Therefore the safety of substituting pegaspargase for a single dose of L-asparaginase in an older population (>60 years of age) must be further evaluated. Of note, a study comparing CLAG-M to standard “7 + 3” induction therapy in patients who progressed to AML from myelodysplastic syndrome reports no significant differences in adverse events, and significantly shorter durations of hospitalizations, with CLAG-M in a patient population that was largely older than 60 years [11]. Consequently, the available data suggest that consideration should be given to using CLAG-M in preference to HAM-pegA in patients >60 years of age.

We acknowledge the limitations of this study. This is a retrospective study and it is possible that presence or severity of adverse events was not adequately documented for data retrieval. The retrospective nature of this study also meant that this was a heterogeneous patient population at baseline, though our pre-treatment demographics were evenly distributed. Patients with a history of venous thromboembolism, pancreatitis, and liver disease may have been excluded from receiving HAM-pegA given the known toxicities of pegaspargase. Admittedly, we also have low power for comparing baseline characteristics statistically. Additionally, whereas the original studies of CLAG-M allowed for two cycles of induction prior to response assessment, in our present study we evaluated responses after only one cycle of induction. Lastly, with the more recent advances in R/R AML regarding targeted therapies for markers such as IDH1/2, FLT3, and CD33, it is acknowledged that some patients may benefit from targeted therapies rather than the presently studied cytotoxic regimens. Future studies would be beneficial to evaluate targeted therapies such as these in combination with a HAM-pegA backbone.

## 5. Conclusions

In conclusion, patients with relapsed or refractory AML treated with HAM-pegA as first salvage cytotoxic therapy had significantly higher response rates and longer event-free survival than patients treated with CLAG-M, while toxicities were similar. Pegaspargase dosed at 2500 units/m^2^ was an effective substitution for L-asparaginase in the HAM-A regimen, with a manageable toxicity profile.

## Figures and Tables

**Table 1:** Baseline Characteristics.

	HAM-pegA *n* (%)	CLAG-M *n* (%)	*p*-Value
Age (years)—median (range)	49 (28–61)	52 (20–67)	NS
Female sex	3 (30.0)	16 (47.1)	NS
Race			0.034 *
White	5 (50.0)	20 (60.6)	
Black	2 (20.0)	12 (36.4)	
Asian	3 (30.0)	1 (3.0)	
Other	-	1 (3.0)	
ECOG			NS
≤2	10 (100.0)	27 (79.4)	
>2	-	4 (11.8)	
Missing data	-	3 (8.8)	
Key Inclusion Criteria			NS
Primary refractory	2 (20.0)	14 (41.2)	
Relapsed disease	8 (80.0)	20 (58.8)	
FAB-Subtype			NS
M0	-	1 (2.9)	
M1	1 (10.0)	4 (11.8)	
M2	3 (30.0)	9 (26.5)	
M4	2 (20.0)	6 (17.6)	
M5	3 (30.0)	7 (20.6)	
M6	-	1 (2.9)	
Missing data	1 (10.0)	6 (17.6)	
Treatment-related AML	-	4 (11.8)	NS
Previous Chemotherapy Received			
7 + 3	9 (90.0)	29 (85.3)	NS
High dose cytarabine	8 (80.0)	18 (52.9)	
Decitabine or Azacitidine	1 (10.0)	7 (20.6)	
CLAG-M	-	1 (2.9)	
Other non-cytotoxic therapy	2 (20.0)	5 (14.7)	
Previous allo-HSCT	3 (30.0)	5 (14.7)	NS
No. Previous CR			NS
1	6 (60.0)	18 (52.9)	
2	2 (20.0)	1 (2.9)	
Unknown	-	1 (2.9)	
Bone marrow blasts—median (range)	33 (4–65)	40 (5–88)	NS
Cytogenetics			NS
Normal karyotype	5 (50.0)	12 (35.3)	
1–2 karyotype abnormalities	3 (30.0)	8 (23.5)	
Complex karyotype	2 (20.0)	14 (41.2)	
Next-generation sequencing available ^^^	6 (60.0)	17 (50.0)	NS
Mutations ^^^—n			
ASXL1	1	3	NS
c-KIT	-	1	
CEBPA	1	1	
FLT3-ITD	2	4	
FLT3-TKD	2	3	
IDH1	-	2	
IDH2	1	-	
NPM1	1	3	
RUNX1	2	6	
TP53	-	2	

FAB = French-American-British classification of acute myeloid leukemia; * Denotes significance, *p* < 0.05; NS = not significant, ^^^ Next-generation sequencing to detect molecular abnormalities was not available on all patients. Molecular abnormalities are therefore reported as number of patients only.

**Table 2:** Treatment Outcomes.

	HAM-pegA	CLAG-M	*p*-Value
**Primary Endpoint**			
Composite complete response (CR + CRh + CRi)	90.0% (9/10)	47.1% (16/34)	0.027
CR	70.0% (7/10)	35.3% (12/34)	NS
CRh	0% (0/10)	0% (0/34)	NS
CRi	20.0% (2/10)	11.8% (4/34)	NS
**Secondary Endpoints**			
Incidence of proceeding to allogeneic HSCT	50.0% (5/10)	26.5% (9/34)	NS
LOS (days)—median (range)	30 (24–37)	35.5 (7–106)	NS
6-month re-hospitalizations—mean	1.5	1.06	NS
30-day survival (%)	100.0	94.1	NS
60-day survival (%)	100.0	88.2	NS

LOS = length of stay; CRh = Complete remission with partial count recovery; CRi = Complete remission with incomplete count recovery; NS = not significant.
